# Association of SNPs in GC and CYP2R1 with total and directly measured free 25-hydroxyvitamin D in multi-ethnic postmenopausal women in Saudi Arabia

**DOI:** 10.1016/j.sjbs.2021.04.071

**Published:** 2021-05-04

**Authors:** Shatha Alharazy, Muhammad Imran Naseer, Eman Alissa, M. Denise Robertson, Susan Lanham-New, Mohammad H. Alqahtani, Adeel G. Chaudhary

**Affiliations:** a Department of Physiology, Faculty of Medicine, King Abdul-Aziz University, Jeddah, Saudi Arabia; b Centre of Excellence in Genomic Medicine Research, King Abdulaziz University, Jeddah, Saudi Arabia; c Department of Medical Laboratory Technology, Faculty of Applied Medical Sciences, King Abdulaziz University, Jeddah 21589, Saudi Arabia; d Department of Clinical Biochemistry, Faculty of Medicine, King Abdulaziz University, Jeddah, Saudi Arabia; e Department of Nutritional Sciences, Faculty of Health and Medical Sciences, University of Surrey, United Kingdom; f Centre for Innovation in Personalized Medicine, King Abdulaziz University, Jeddah, Saudi Arabia

**Keywords:** Vitamin D, Vitamin D binding protein, *GC*, *CYP2R1*, Single nucleotide polymorphism, Saudi Arabia

## Abstract

**Background:**

Group-specific component (*GC*) and cytochrome P450 Family 2 Subfamily R Member 1 (*CYP2R1*) genes are one of the vital genes involved in the vitamin D (vitD) metabolic pathway. Association of genetic polymorphisms in these two genes with 25-hyroxyvitamin D (25(OH)D) level has been reported in several studies. However, this association has been reported to be discrepant among populations from different ethnicities. Therefore, we aimed in this study to investigate association of the two major single nucleotide polymorphisms (SNP) in *GC* (rs4588 and rs7014) and a SNP (rs12794714) in *CYP2R1* in postmenopausal women in Saudi Arabia.

**Methods:**

This study randomly selected 459 postmenopausal women (aged ≥50 years) of multiple ethnicities in Jeddah, Saudi Arabia. Blood samples were collected from all participating women for DNA extraction and for assessment of serum levels of total 25(OH)D, directly measured free 25(OH)D and other biochemical parameters. SNPs in selected vitD related genes (rs4588 in *GC*, c.1364G > T with transcript ID: NM_001204307.1 and rs7041 in *GC*, c.1353A > C with transcript ID NM_001204307.1 and rs12794714 in *CYP2R1*, c.177G > A with transcript ID NM_024514.4) were determined in DNA samples using Sanger DNA sequencing.

**Results:**

Minor allele frequency for rs4588, rs7041 and rs12794714 were 0.25, 0.44 and 0.42 respectively. Genotypes of rs7041 showed significant difference in total 25(OH)D level but not in free 25(O)D level (P = 0.023). In comparison, genotypes of rs4588 and rs12794714 did not show any significant difference neither in total nor in free 25(OH)D level. Post hoc test revealed that total 25(OH)D was lower in the rs7041 TT allele compared to the GG allele (P = 0.022). Chi-square test showed that vitD status was associated with rs7041 genotypes (P = 0.035). In addition, rs7041 minor alleles were found to have an association with vitD deficiency with a statistical significant odds ratio (>1) of 2.24 and 3.51 with P = 0.006 and P = 0.007 for TG and GG genotypes respectively.

**Conclusion:**

The rs7041 SNP in *GC* was associated with total 25(OH)D level in postmenopausal women in Saudi Arabia, while rs4588 in *GC* and rs12794714 in *CYP2R1* did not show association with total 25(OH)D. Further studies exploring additional variants in vitD related genes are needed to understand genetic factors underlying vitD deficiency in Saudi population.

## 1 Introduction

Vitamin D (VitD) plays an important role in numerous human metabolic functions including calcium (Ca) and phosphate (PO4) hemostasis and bone growth and remodeling (Holick, 2007, Holick et al., 2011). VitD sufficiency is not only vital for skeletal function but may be also related to prevention of several pathological conditions including cardiovascular diseases, malignancies, autoimmune diseases and as well as severity of COVID-19 (Holick, 2004, Laird et al., 2020). Genetic studies related to vitD have reported several genetic variants or SNPs that could influence 25(OH)D status (Jolliffe et al., 2016, McGrath et al., 2010a, McGrath et al., 2010b). SNPs can result in a decrease or increase in the serum concentration of 25(OH)D depending on its effect on the translation of enzymes, binding proteins or receptors (McGrath et al., 2010a, McGrath et al., 2010b).

VitD, whether it is sun-derived (vitD3) or dietary (vitD3 and ergosterol-derived vitD2 taken form food or supplements) is hydroxylated in the liver mainly by cytochrome P450 enzyme (25-hydroxylase encoded by the *CYP2R1* gene) to form 25(OH)D (DeLuca et al., 1971, Shinkyo et al., 2004). Metabolites of vitD including 25(OH)D and 1,25-dihydroxyvitamin D (1,25(OH)_2_D), are mainly transported by vitamin D binding protein (VDBP) encoded by the *GC* gene (Daiger et al., 1975, Speeckaert et al., 2006). Although there are several forms of cytochrome *p*-450, *CYP2R1* has the greatest specificity and affinity to vitD (Schuster, 2011) with a missense mutation in *CYP2R1* reported to cause vitD deficiency (Cheng et al., 2004). In addition, a number of genome-wide association studies in cohorts from European descent detected more than 25 SNPs in *CYP2R1* linked with vitD status (Ahn et al., 2010, Anderson et al., 2014, Jiang et al., 2018, O'Brien et al., 2018, Wang et al., 2010). However, several studies performed in populations from non-European cohorts have been unable to replicate these results (Duan et al., 2018, Lu et al., 2012, Robien et al., 2013, Xu et al., 2015).

To date, 25(OH)D is considered as the major vitD metabolite representing vitD status (Del Valle et al., 2011, Hollis, 1996). Circulating 25(OH)D is bound mainly to VDBP (85–90%), around 10 to 15% is bound weakly to albumin, with only a small fraction (0.03%) as free and unbound (Bikle et al., 1986). Based on the “free hormone hypothesis”, free 25(OH)D is the only form of 25(OH)D that can pass into the cells and process their biological actions (Bikle et al., 1986) so, free 25(OH)D is suggested to be a better surrogate marker for evaluating vitD status than the total 25(OH)D (Chun et al., 2014, Johnsen et al., 2014). Free 25(OH)D can be measured either by means of a direct method, using a recently developed immunoassay, or estimated by calculations using the concentrations of albumin, VDBP and total 25(OH)D (Bikle et al., 1986, Lee et al., 2015).

VDBP is an extremely polymorphic protein with different alleles that have considerable effect on its biological functions (Arnaud and Constans, 1993, Constans et al., 1985). The different degrees of VDBP affinity to 25(OH)D has been proposed to influence both VDBP and 25(OH)D concentrations (Arnaud and Constans, 1993, Constans et al., 1985). Common genetic polymorphisms in the *GC* gene that encodes for VDBP result in the production of different proteins with different affinities to vitD (Bhan, 2014). The most common SNPs in *GC* reported in the community are r4588 and rs7041 (Braun et al., 1992, Dastani et al., 2013). These polymorphisms are related to ethnicity, are highly prevalent and associated with the functional role of VDBP (Arnaud and Constans, 1993, Braun et al., 1992, Constans et al., 1985, Engelman et al., 2008). Genetic discrepancies in VDBP in populations of different ethnic background result in different levels of total 25(OH)D (Fischer, 2020, Powe et al., 2013). For example, Africans have VDBP variants associated with lower VDBP binding affinity to 25(OH)D and lower levels of total 25(OH)D when compared with Caucasians even if both racial groups have similar free 25(OH)D levels (Aloia et al., 2015, Powe et al., 2013). As vitD genetic studies particularly in *GC* and *CYP2R1* have shown divergence in their results across populations from different ethnic backgrounds, and as vitD deficiency is an extremely prevalent issue in Saudi Arabia with residents in the western region of Saudi Arabia (Jeddah) coming from multiple ethnic groups. We aimed in this study to investigate the prevalence of the two most common SNPs in *GC* (rs4588 and rs7041) and a SNP in *CYP2R1* (rs12794714) and to study their association with vitD status including total 25 (OH)D, directly measured free 25 (OH)D and VDBP in a postmenopausal cohort in Saudi Arabia.

## 2 Methods

### 2.1 Study design and recruitment

This study included a total of 459 postmenopausal women (aged ≥50 years) in Jeddah, Saudi Arabia. We assessed in the participants vitD status (levels of total 25(OH)D, directly measured free 25(OH)D and VDBP) and its association with vitD related genes SNPs (rs4588 and rs7041 in *GC* and rs12794714 in *CYP2R1*). In addition, participants were subcategorized according to their ethnic origin. To ensure that the participating women represent a randomly-selected adult population, participants were recruited from seven primary health care centers located across Jeddah in which each represented one of the seven geographical divisions of the city. Women who agreed to participate were referred to a clinic at the Centre of Innovation in Personalized Medicine (CIPM) in King Fahad Medical Research Centre (KFMRC), King Abdulaziz University (KAU), Jeddah. Written informed consent for participation in the study was provided by each participant. Ethical standards of Declaration of Helsinki were followed throughout this study and a favourable ethical opinion was provided by the Research Ethics Committee in the Unit of Biomedical Ethics, Center of Excellence in Genomic Medicine Research (CEGMR), KAU, (05-CEGMR-Bioeth-2018).

Postmenopausal status for this study was defined as the ceasing of menses for a period of one year and above, with a level of serum follicular stimulating hormone (FSH) greater than 15mIU/m. Exclusion criteria included history of chronic renal and liver disease, cancer, rheumatoid arthritis, malabsorption syndrome, hyperthyroidism, hyperparathyroidism, diabetes or intake of medications with potential influence on vitD levels (e.g. glucocorticoids and anticonvulsants). Women with high levels of creatinine and liver enzymes were excluded (the normal clinical level of serum creatinine in females < 105 μmol/L; Aminotransferase (AST) < 45 U/L; Alanine Aminotransferase (ALT) < 50 U/L and Alkaline Phosphatase (ALP) 80–280 U/L). Women with thyroid stimulating hormone (TSH) levels below 0.465 mIU/L were also excluded.

### 2.2 Study procedure and blood analysis

Each participant underwent basic anthropometric measurements. Blood samples were also collected in tubes containing ethylenediaminetetraacetic acid (EDTA) as an anticoagulant (for DNA) and plain tubes containing no additives (no clot activators, anticoagulants, preservatives or separator material) for serum from all participants. The following parameters were measured in all samples, as serum total serum 25(OH)D and intact PTH quantified by chemiluminescence immunoassay (CLIA), using a LIAISON auto-analyzer (DiaSorin Inc., Stillwater, MN, USA), directly measured free 25 (OH)D by immunoassay using ELISA kit (KAPF1991, Future Diagnostics Solutions B.V., Wijchen, Netherlands), VDBP by quantitative sandwich enzyme immunoassay technique using Quantikine® ELISA (DVDBP0B, R&D Systems, Minneapolis, MN, USA). Serum albumin, Ca, PO_4_, magnesium (Mg), lipid profile and blood glucose were measured by the colorimetric method using a VITROS 250 Clinical Chemistry auto-analyzer (Ortho-Clinical Diagnostics Inc., Rochester, NY, USA).

### 2.3 Genetic analysis

Genomic DNA was first extracted using a DNA extraction kit (53104, Qiagen, Hilden, Germany). The concentration and purity of the DNA filtrate was measured by NanoDrop spectrophotometer (ND-1000 UV–VIS). To screen for SNPs in selected vitD related genes (rs4588 and rs7041 in *GC* and rs12794714 in *CYP2R1*), bespoke targeted primers were designed using web-based Primer3 (v. 0.4.1) software. The forward primer for *GC* was 5′-TCATTGCAAAGACAGCCAAGT-3′ and reverse primer was 5′-GACTTCCAATTCAGCAGCGA-3′. The forward primer for *CYP2R1* was 5′-AAATCAGGACTGGATCGCC-3′ and reverse primer was 5′-CAATGGGAGTATGGCAGGGC-3′. Synthesized primers (Macrogen Inc, Seoul, Korea) were prepared by adding appropriate volume of nuclease-free water to the vial of each powder primer to form concentration of 100 pmol/μl, then each vial was vortexed and mixed properly. Next, aliquots of primers for polymerase chain reaction (PCR) were prepared by mixing 10 μl of the stock of each primer with 90 μl of nuclease-free water to form concentration of 10 pmol/μl. Aliquots of primers for sequencing were prepared by adding 96.8 μl of nuclease-free water to 3.2 μl of stock primer to form a concentration of 3.2 pmol/μl. Two μl of DNA samples, 1 μl of forward and reverse primer along with 10 μl GoTaq® Green Master mix (M7123, Promega, WI, USA) and 11 μl nuclease free water were mixed and vortexed inside the PCR tube. Afterwards, PCR tubes were spun down and placed in thermal cycler (VERITI 96, Applied Biosystems, Thermo Fisher Scientific, Waltham, MA, USA) to proceed with touchdown PCR. For the Sanger sequencing method, four steps were achieved: purification of PCR products, cycle sequencing reaction, purification of cycle sequencing products, and denaturation and samples’ loading into the sequencer.

PCR amplification and purification were performed for DNA samples using a PCR purification kit then Sanger sequencing was conducted using a genetic analyzer (3500 genetic analyzer, Applied Biosystems, Thermo Fisher Scientific, Waltham, MA, USA) and BigDye Terminator V3.1 Cycle Sequencing kit (cat#4337455, Applied Biosystems, ThermoFisher Scientific, MA, USA). These technologies afford sequence reads of 400–700 nucleotides and consequently the target variants are simply observed using ABI genetic analyzers. Sanger sequencing data were elicited in ABI sequence trace format files. Every sequence trace was lined up with the analogous reference sequence using BioEdit software (version 7.2.5) that uses the fluorescence intensity at every data point to call for each base.

### 2.4 Statistical analysis

The results of the study were statistically analyzed using SPSS program (v.20 SPSS Chicago Inc, 2011). Normality of data was examined by Kolmogorov-Smirnov test. Medians and interquartile ranges (IQR) were calculated for numerical non-parametric data, while means and standard deviations (SD) were calculated for numerical parametric data. Descriptive data were presented as a percentage of the total sample number. Comparison between groups for non-normally distributed data was performed using Kruskal-Wallis H test. Post hoc test (Dunn’s test) was used to identify which groups were different. The Fisher Freeman-Halton exact test was used to determine the relationship between categorical variables with expected cell count < 5 while chi-square test of independence was used with expected cell count ≥5. The association between the studied SNPs (exposure) and vitD deficiency (outcome) was measured by Odds ratio. A probability value ≤0.05 was considered statistically significant.

## 3 Results

### 3.1 General and genotypic features of the subjects

General characteristics of the participants are shown in Table 1 including biochemical parameters. The majority (88%) of the participating postmenopausal women were whites from Arabic origins while 9% were originally black Africans and 3% were of Pakistani-South Asian descent. Median (IQR) for levels of total 25(OH)D, directly measured 25(OH)D and VDBP were 19.9 (12.2–29.6) ng/ml, 4.69 (3.58–6.45) pg/ml and 353 (207–616) μg/ml, respectively.

**Table 1:** Baseline general characteristics of all participating women.

Variable	Results (n = 459)
Age (years)	58 (54–64)
Years since menopause	7 (3–15)
BMI (kg/m^2^)	31.4 ± 6.6
Ethnicity:	
White (Arabic)	263 (88%)
Black (African)	28 (9%)
South Asian (Pakistani)	11 (3%)
Serum Total 25(OH)D (ng/ml)	19.9 (12.2–29.6)
Serum direct free 25(OH)D (pg/ml)	4.69 (3.58–6.45)
Serum VDBP (μg/ml)	353 (207–616)
Serum Intact PTH (pg/ml)	21.8 (13.6–36)
Serum Albumin (g/L)	44 (40–48)
Serum Ca (mmol/L)	2.4 (2.37–2.44)
Serum PO_4_ (mmol/L)	1.36 (1.25–1.49)
Serum Mg (mmol/L)	0.8 (0.8–0.9)
Serum HDL-C (mmol/L)	1.40 (1.10–1.60)
Serum LDL-C (mmol/L)	3.06 (2.54–3.60)
Serum VLDL-C (mmol/L)	0.58 (0.43–0.75)

Data are described as mean ± SD with normal distribution and as median (IQR) with non-normal distribution. Descriptive data are presented as n (%). (%) is percentage out of the total number of subjects. BMI is Body Mass Index; 25(OH)D is 25-hydroxyvitamin D; VDBP is vitamin D binding protein; PTH is parathyroid Hormone; Ca is calcium; PO4 is phosphate; and Mg is magnesium; HDL-C is high density lipoprotein cholesterol; LDL-C is low density lipoprotein cholesterol; VLDL-C is very low density lipoprotein cholesterol.

**Fig. 1:**
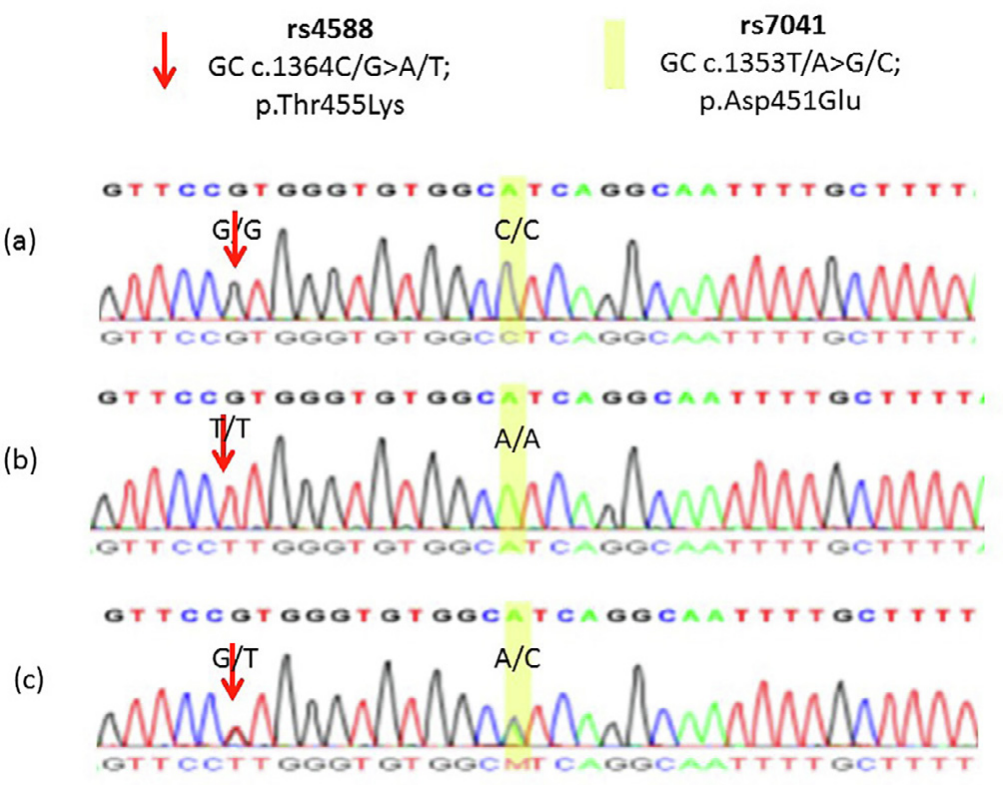
Samples of observed Sanger sequencing chromatograms that showed rs4588 and rs7041 SNPs in GC gene in the participating women (n = 459). **(a)** Chromatogram is showing G/G homozygous as a reference genotype for rs4588, while C/C homozygous replacing A/A for rs7041. **(b)** Chromatogram is showing homozygous T/T replacing G/G for rs4588, whereas homozygous A/A as a reference genotype for rs7041. **(c)** Chromatogram is showing heterozygous G/T and A/C for rs4588 and rs7041, respectively.

**Fig. 2:**
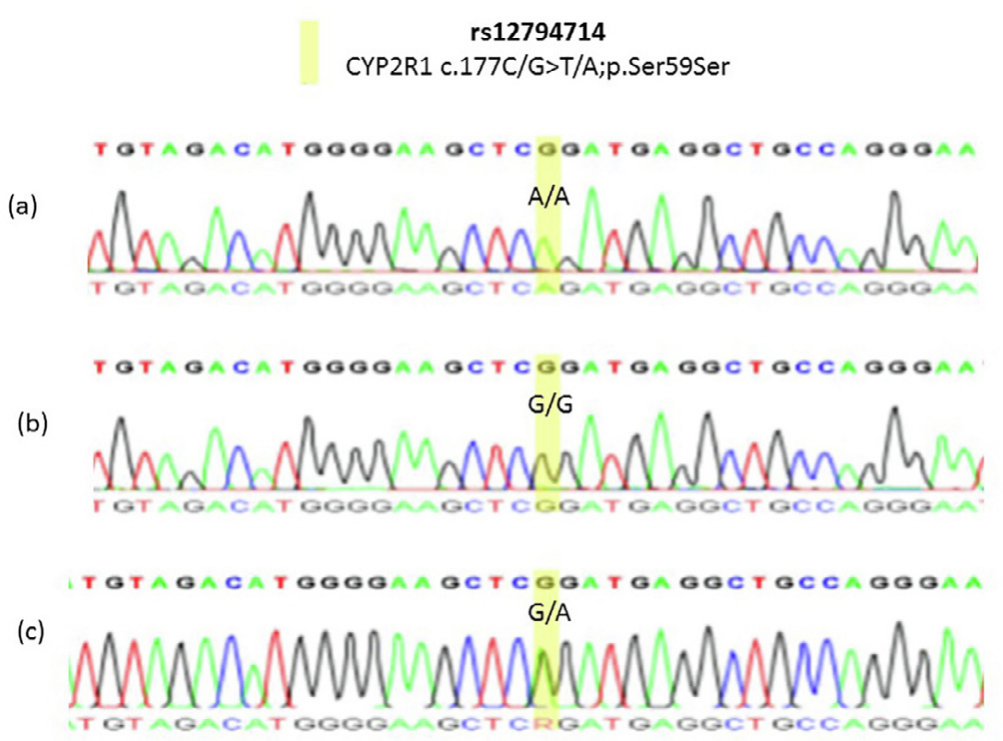
Samples of observed Sanger sequencing chromatograms that showed rs12794714 SNP in CYP2R1 gene in the participating women (n = 459). **(a)** Chromatogram is showing a homozygous A/A replacing G/G for rs12794714. **(b)** Chromatogram is showing homozygous G/G as a reference genotype for rs12794714. **(c)** Chromatogram is showing heterozygous G/A for rs12794714.

Fig. 1, Fig. 2 show examples of observed Sanger sequencing chromatogram results of SNPs in *GC* (rs4588, c.1364G > T with transcript ID: NM_001204307.1 and rs7041, c.1353A > C with transcript ID NM_001204307.1) and a SNP in *CYP2R1* (rs12794714, c.177G > A with transcript ID NM_024514.4). Chromatograms showed homozygous G/G and A/A as reference genotypes for rs4588 and rs7041, respectively. They also showed homozygous T/T replacing G/G for rs4588 and homozygous C/C replacing A/A for rs7041. Heterozygous G/T and A/C were observed for rs4588 and rs7041, respectively. For rs12794714, chromatograms showed homozygous G/G as a reference genotype, homozygous A/A replacing G/G and heterozygous G/A.

**Fig. 3:**
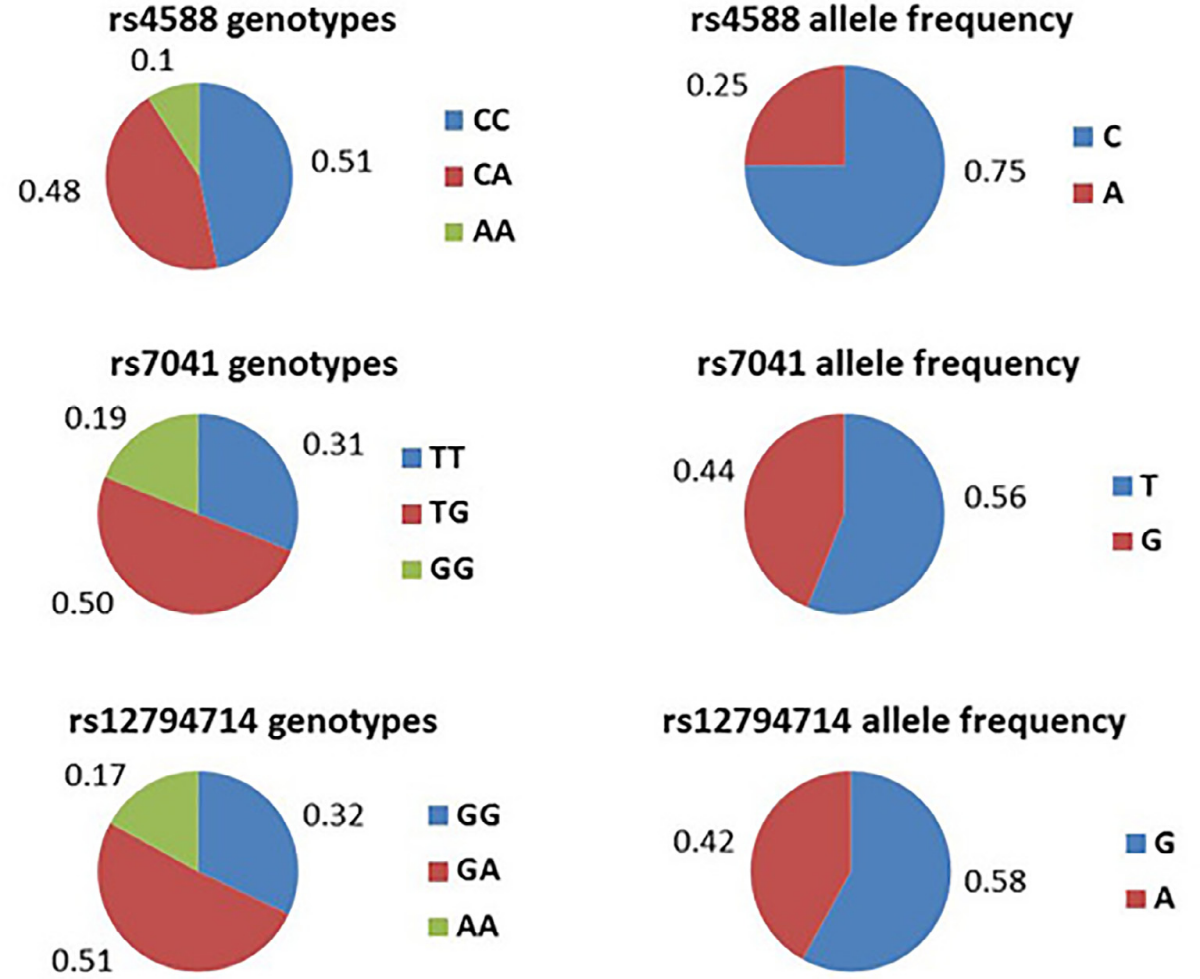
Genotypes and alleles frequencies of rs4588, rs7041 and rs12794714 among postmenopausal participating women (n = 459).

The frequencies of genotypes and alleles of the two SNPs in *GC* (rs4588 and rs7041) and rs12794714 SNP in *CYP2R1* in the participating women are shown in Fig. 3. Fifty one percent of the study cohort were genotyped as CC for rs4588 and GA for rs12794714, and 50% of the participants were genotyped as TG for rs7014. The frequency of the minor allele for rs4588 and rs7041 were 0.25 and 0.44, respectively. The minor allele frequency for rs12794714 was 0.42.

**Fig. 4:**
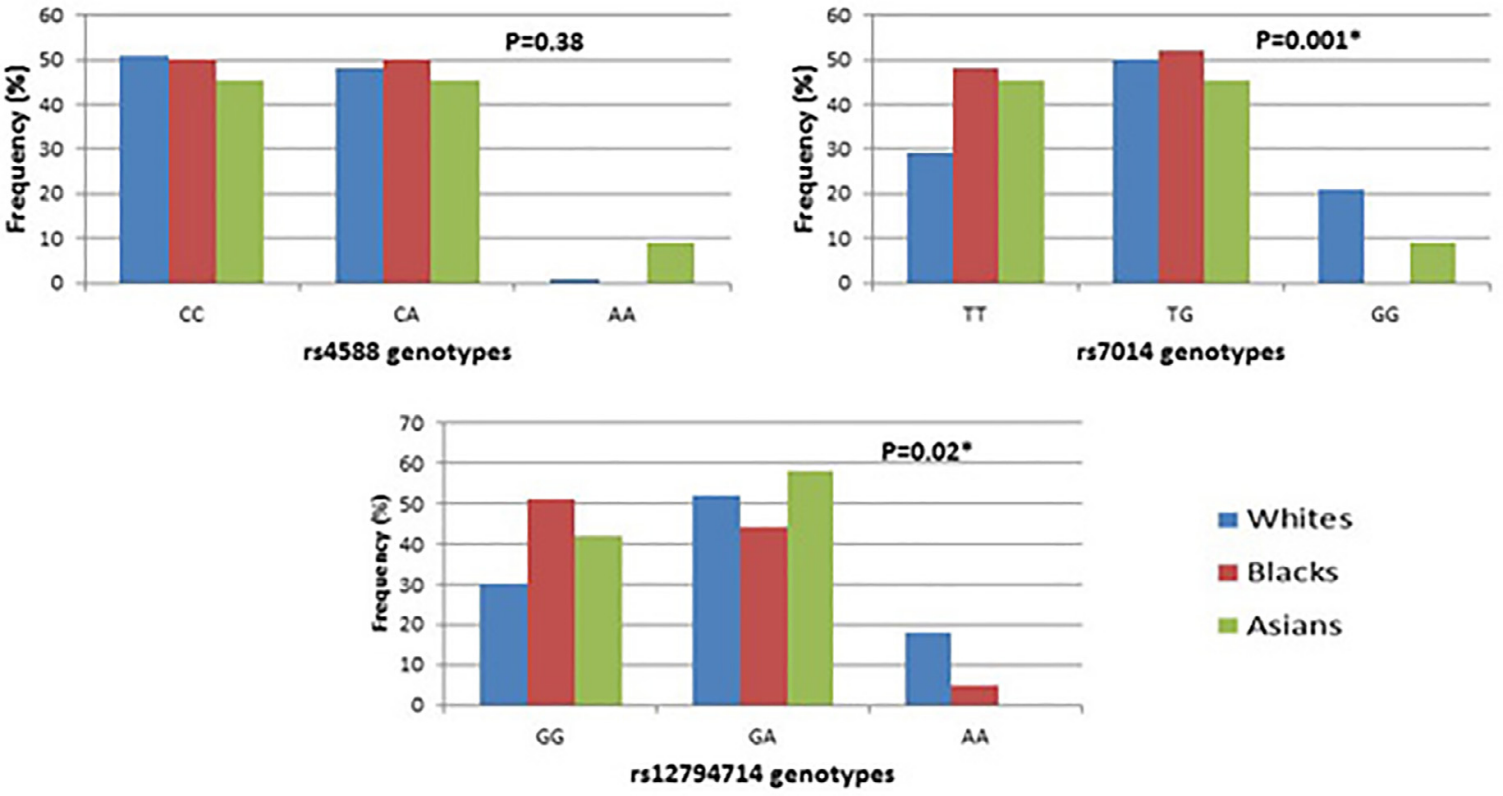
Racial differences in the frequency of the studied vitD related SNPs. Fisher Freeman-Halton exact test was used to determine the relation of ethnicity with the studied SNPs (*Significant correlation; P < 0.05).

The prevalence of the studied SNPs genotypes among the subcategorized ethnic groups (black, white, Asian) is displayed in Fig. 4. Frequency of rs7041 and rs12794714 genotypes showed statistically significant difference between ethnic groups (P = 0.001 and P = 0.02, respectively).

### 3.2 Association between genotypes and vitamin D status (levels of total and free 25(OH)D)

Total and directly measured free 25(OH)D and VDBP levels in the different SNPs genotypes groups are shown in Table 2. Genotypes of rs7041 showed significant differences in total 25(OH)D level but not in free 25(O)D level (P = 0.023). In comparison, genotypes of rs4588 and rs12794714 did not show any significant differences in total or free 25(OH)D level. Post hoc testing revealed that total 25(OH)D was different (lower) in the rs7041 TT allele compared to the GG allele (P = 0.022).

**Table 2:** Association of free and total 25(OH)D and VDBP with the studied SNPs genotypes.

SNP (Gene)	Genotype (n = 459)	Total 25 (OH)D level (ng/ml)	P-value	Free 25 (OH)D level (pg/ml)	P-value	VDBP (μg/ml)	P-value
**rs4588 (*GC*)**	CC	21.8 (13.5–31.7)	0.059	0.023 (0.017–0.03)	0.98	361 (203–679)	0.34
	CA	19 (11.3–26)		0.027 (0.019–0.035)		342 (2.2–563)	
	AA	19.2 (17–27.6)		0.026 (0.16–0.43)		297 (219–394)	

**rs7041 (*GC*)**	TT	18.1 (10.4–26.7)	0.023*	4.9 (3.6–6.5)	0.85	309 (198–526)	0.14
	TG	20.6 (13.1–29.1)		4.5 (3.4–6.2)		409 (214–675)	
	GG	23.1 (14.1–31.7)		5.04 (3.9–6.9)		347 (189–750)	

**rs12794714 (*CYP2R1*)**	GG	20.5 (11.4–30.7)	0.24	4.9 (3.6–6.5)	0.33	357 (204–600)	0.76
	GA	20.7 (13.2–30.8)		4.7 (3.8–6.6)		347 (204–633)	
	AA	21.4 (13.2–39.8)		4.04 (3.3–6)		354 (189–563)	

* Significant correlation (P < 0.05). Median (IQR) differences in 25(OH)D and VDBP between SNPs categories were determined using Kruskal-wallis test.

When vitD status categories were correlated with SNPs genotypes using chi-square test, vitD status was associated with rs7041 genotypes (P = 0.035). In addition, rs7041 minor alleles were found to have an association with vitD deficiency with a statistical significant odds ratio (>1) of 2.24 and 3.51 with P = 0.006 and P = 0.007 for TG and GG genotypes respectively (Table 3).

**Table 3:** Association between vitD status and studied SNPs genotypes in all participants.

SNP (Gene)	Genotype (n = 459)	VitD deficient (n = 111) n (%)	VitD insufficient (n = 125) n (%)	vitD sufficient (n = 223) n (%)	Odds ratio* (95% CI)	P-value	Chi-square (P-value)
**rs4588 (*GC*)**	CC (n = 232)	47 (42%)	62 (50%)	121 (54%)	1 (Ref)	–	(0.15)♦
	CA (n = 222)	64 (58%)	59 (47%)	100 (45%)	1.15 (0.63–2.10)	0.65	
	AA (n = 6)	0 (0%)	4 (3%)	2 (1%)	–	–	

**rs7041 *(GC*)**	TT (n = 141)	49 (44%)	38 (30%)	60 (27%)	1 (Ref)	–	10.32 **(0.035)**
	TG (n = 231)	48 (43%)	66 (53%)	114 (51%)	2.24 (1.25–4.0)	**0.006**	
	GG (n = 87)	14 (13%)	21 (17%)	49 (22%)	3.51 (1.4–8.8)	**0.007**	

**rs12794714 *(CYP2R1*)**	GG (n = 147)	41 (37%)	35 (28%)	74 (33%)	1 (Ref)	–	6.018 (0.198)
	GA (n = 234)	48 (43%)	65 (52%)	120 (54%)	1.35 (0.78–2.33)	0.29	
	AA (n = 78)	22 (20%)	25 (20%)	29 (13%)	0.82 (0.41–1.65)	0.58	

* Odd ratios for vitD deficiency were compared with the homozygous genotype in the reference genome.

♦ Fisher Freeman-Halton exact test was used as 3 cells in rs4588 had expected count <5.

## 4 Discussion

In our study population, the minor allele frequency of rs7041(G/T) was 0.44 which is identical to the frequency reported in a GWAS by Wang et al. (Wang et al., 2010) and was close to that reported in both Kuwaiti Arabs (0.43) (Elkum et al., 2014) and Canadian Europeans (0.41) (Gozdzik et al., 2011), but lower than that reported in Hispanics (0.59) (Engelman et al., 2008). In contrast, the minor allele frequency of rs4588(C/A) was 0.25 which was similar to that reported in Hispanics (0.25) (Engelman et al., 2008), South Brazilian (0.26) (Santos et al., 2013) and Canadian Europeans (0.28) (Engelman et al., 2008) but different to Arab Jordanians (0.19) (Lafi et al., 2015). In addition, the minor allele frequency of rs12794714(G/A) in our study **(**0.42) was comparable to that found in Kuwaiti Arabs (0.42) (Elkum et al., 2014) and Europeans (0.43) (Wang et al., 2010), and minimally different to that found in Chinese populations (0.37) (Xu et al., 2014). This discrepancy in minor allele frequencies can be explained by the role of ethnicity in changing the distribution of these alleles among different populations as observed in our study that there was an ethnic variation in frequency distribution of rs7041 and rs12794714 genotypes among white, black and Asian ethnic subgroups.

One of the aims of this research was to investigate the relationship of vitD status (including free and total 25(OH)D level and VDBP level) with the two common SNPs in *GC* (rs4588 and rs7041), and the SNP in *CYP2R1* (rs12794714). We found that the rs7041 SNP in the *GC* gene, which encodes for VDBP (the major carrier of vitD) (Daiger et al., 1975, Speeckaert et al., 2006), was associated with total 25(OH)D level and vitD status, with the TT genotype of rs7041 being associated with lower vitD level compared to the GG genotype. This observation is in accordance to what was found previously in the Arab and South Asian populations, in which total 25(OH)D was associated with rs7041 polymorphism (Elkum et al., 2014, Lafi et al., 2015, McGrath et al., 2010a, McGrath et al., 2010b). This also confirms finding of previous studies in Europeans that have shown that rs7041T and rs4588A are associated with decreased blood 25(OH) levels (Berry and Hyppönen, 2011, Dastani et al., 2013, Powe et al., 2013, Wang et al., 2010). However, our study did not find such an association. Noticeably, our study included only women, and it is suggested that the genetic influence on vitD concentration might be stronger in males compared to females (Arguelles et al., 2009) which might explain why this study only reported a trend (p = 0.059) when rs4588 was correlated with total 25(OH)D concentration.

Concerning the studied SNP rs12794714 in *CYP2R1*, the gene responsible for second step of vitD activation in the liver (DeLuca et al., 1971, Shinkyo et al., 2004), we did not find any association with this SNP and 25(OH)D levels which is consistent to studies in Chinese populations (Lu et al., 2012, Xu et al., 2014). Conversely, several studies in white Europeans have reported an association between *CYP2R1* polymorphisms and 25(OH)D level (Ahn et al., 2010, Bu et al., 2010, Wang et al., 2010). This disagreement can be attributed to role of ethnicity in influencing relationship of 25(OH)D with genetic polymorphisms (Powe et al., 2013).

Data on genetic determinants of free 25(OH)D is lacking. A previous study did not find any significant association between calculated free 25(OH)D levels and several SNPs in major genes involved in vitD metabolism including rs4588 and rs7041 (Szili et al., 2018). Equivalently, our study was not able to find any association between free 25(OH)D and the investigated SNPs. This suggests that total 25(OH)D level might be more genetically influenced than free 25(OH)D level through genetic variations in GC such as rs7041.

Given the fact that *GC* variants play a role in influencing levels of VDBP and its affinity to vitD thus influencing vitD status (Arnaud & Constans, 1993), it is not surprising that total 25(OH)D was associated with the rs7041 SNP in VDBP. Our finding adds more evidence on rs7041 association with 25(OH)D level. It confirms that populations in Saudi Arabia where the majority of the cohort are from Arabic origin is aligned with other populations of different ethnicities including Europeans in terms of rs7041 association with 25(OH)D level (Berry and Hyppönen, 2011, Dastani et al., 2013, Powe et al., 2013, Wang et al., 2010). Whether VDBP genetic polymorphisms including rs7041 changes the response of vitD levels to vitD supplementation and sunlight exposure needs to be addressed in further studies. This study has revealed an aspect of genetic variation influencing vitD level in the population of Saudi Arabia. It included only healthy postmenopausal women to exclude the adverse influence of diseases and medications on vitD levels, which is a strength of this study. Nevertheless, there are number of limitations which should be highlighted including the small number of studied SNPs and the uncontrolled confounding factors affecting vitD status such as age, obesity and sunlight exposure.

## 5 Conclusion

The current study confirmed a significant association between the *GC* SNP (rs7041) and total 25(OH)D level in postmenopausal women in Saudi Arabia. VitD deficiency seems to be associated with rs7041. Conversely, rs4588 in *GC* and rs12794714 in *CYP2R1* did not show significant associations with total 25(OH)D. Further studies exploring rs7041 in larger-scale populations with additional variants in vitD related genes are required in Saudi Arabia, which could provide a new insight into the treatment of vitD deficiency and personalized vitD recommendations based on genetic background.

## 6 Ethics approval and consent to participate

Ethical approval of this study was obtained from the Research Ethics Committee in Unit of Biomedical Ethics, CEGMR, KAU (ref no.05-CEGMR-Bioeth-2018). Fully informed, written consent was obtained from the participants.

## 7 Consent for publication

Not applicable.

## 8 Availability of data and materials

The datasets used and/or analysed during the current study are available from the corresponding author on reasonable request.

## Funding

Joint supervision programme, KAU, Jeddah, Saudi Arabia. The funders had no role in study design, data collection and analysis, decision to publish, or preparation of the manuscript.

## 10 Authors' contributions

SA contributed to the study design and execution, data analysis and manuscript drafting. MHQ contributed to study design. MIN contributed in data analysis, writing, editing and review. EA contributed to writing review and supervision. SL-N contributed to supervision. MDR and AGC contributed to editing, review and supervision. All authors read and approved the final manuscript.

## Declaration of Competing Interest

The authors declare that they have no known competing financial interests or personal relationships that could have appeared to influence the work reported in this paper.
